# Establishment, Culture, and Characterization of Guinea Pig Fetal Fibroblast Cell

**DOI:** 10.1155/2014/510328

**Published:** 2014-03-25

**Authors:** Davood Mehrabani, Reza Mahboobi, Mehdi Dianatpour, Shahrokh Zare, Amin Tamadon, Seyed Ebrahim Hosseini

**Affiliations:** ^1^Stem Cell and Transgenic Technology Research Center, Shiraz University of Medical Sciences, 71348 Shiraz, Iran; ^2^Department of Medical Genetics, School of Medicine, Shiraz University of Medical Sciences, 71348 Shiraz, Iran; ^3^Department of Physiology, Islamic Azad University, Marvdasht Branch, 73985 Marvdasht, Iran

## Abstract

Establishment of Guinea pig fetal fibroblast cells and their biological evaluation before and after cryopreservation were the main purposes of this study. After determination of the proper age of pregnancy by ultrasonography, 30 days old fetuses of Guinea pigs were recovered. Their skins were cut into small pieces (1 mm^2^) and were cultured. When reaching 80–90% confluence, the cells were passaged. Cells of the second and eighth passages were cultured in 24-well plates (4 × 10^4^ cells/well) for 6 days and three wells per day were counted. The average cell counts at each time point were then plotted against time and the population doubling time (PDT) was determined. Then, vials of cells (2 × 10^6^ cells/mL) were cryopreserved for 1 month and after thawing, the cell viability was evaluated. The PDT of the second passage was about 23 h and for the eighth passage was about 30 h. The viability of the cultures was 95% in the second passage and 74.5% in the eighth passage. It was shown that the Guinea pig fetal fibroblast cell culture can be established using the adherent culture method while, after freezing, the viability indices of these cells were favorable.

## 1. Introduction

At present, preservation of individual animals, semen, embryos, and genomic and cDNA libraries are all practical options. Traditionally, in vitro conservation includes materials like semen, oocytes, and embryos even for a few numbers of species that require species-specific techniques [1]. Cryopreservation of somatic cells has opened a new option for conserving species [2]. In addition, cloning techniques have made somatic cells as an attractive resource for conserving animal genetic materials [3]. With further development of animal cloning technology, the establishment of a somatic cell bank is extremely important for the recovery of endangered species. For every animal, tissue samples can be cryopreserved in liquid nitrogen and it can be a choice method for the rapid creation of emergency gene banks.

The establishment of fibroblast banks especially for endangered species can provide an excellent resource for biological research and preserve precious genetic materials [4]. Fibroblast cells have been cultured in vitro from several species and tissues and have different applications including nuclear transfer in cloning, feeder layer of embryonic stem cells, wound healing researches, and tissue engineering purposes. Ear marginal tissues or fetal skin tissues were isolated for adherent culture methods to establish fibroblast cell banks. Somatic cell banks have also been established for some breeds such as cattle [5, 6], sheep [7–9], and goat [4, 10–14].

To preserve a valuable genetic resource, establishing fibroblast banks has been proposed as a practical approach; not only does it preserve precious genetic material, but it also provides an excellent resource for biological research. The purpose of this study was to establish and biologically evaluate the fibroblast cultures provided from fetal skin tissue of Guinea pigs.

## 2. Materials and Methods

### 2.1. Fetus Collection and Skin Culture

Mature female Guinea pigs (*Cavia porcellus*) were selected and housed in the Laboratory Animal Center of Shiraz University of Medical Sciences, Shiraz, Iran. The animals were housed in standard cages, three per cage (2 females and 1 male), under controlled temperature (22°C), with a 12 h light and 12 h dark cycle. Standard laboratory chow and tap water were available ad libitum. Four pregnant female Guinea pigs (*Cavia porcellus*) were enrolled and the gestational age was determined ultrasonographically by measuring the fetal biparietal diameter [15] using a real-time B-mode ultrasound scanner (SIUI V900, China) equipped with a 7.5 MHz, linear-array transducer and applying the formula of *Y* = −0.00043*X* ^2^ + 0.06881*X* − 0.75941; where *Y* = gestation day and *X* = biparietal diameter (cm).

The Guinea pigs were anesthetized by an intraperitoneal injection of ketamine (100 mg/kg, Woerden, The Netherlands) and xylazine (7 mg/kg, Alfazyne, Woerden, Netherlands). They were decapitated and their uteruses were removed immediately and washed in sterile phosphate buffered saline (PBS; GIBCO cat. number 18912-014) containing 1% penicillin and streptomycin (Sigma cat. number of P-4687 and S-1277, St. Louis, USA). The skin of fetuses was cut into small pieces (1 mm^2^) and cultured in 88% Dulbecco's Modified Eagle Medium (DMEM; Gibco cat. number 12800-116), 10% fetal bovine serum (FBS; Gibco, cat. number 10270-106), 1% penicillin and streptomycin, and 1% L-glutamine (Sigma cat. number G5840) in 25 cm^2^ flasks and were cultured at 37°C in an incubator with 5% CO_2_ and saturated humidity. The medium was replaced after 48 h. When fibroblast cells reached 80–90% confluence, the cells were harvested using 0.25% trypsin (Gibco cat. number 15090-046) and were then passaged until reaching the eight passages.

### 2.2. Determination of the Proper Age of Fetus for Fibroblast Collection

In the first step, the Guinea pig fetuses were collected after determination of the pregnancy age (days 20, 30, and 40 of pregnancy). The skin of fetuses was cultured as described before and the time intervals between the first and second passages (when the cell cultures reached 80–90% confluence) in the three different ages were determined.

### 2.3. Growth Curve Analysis

In the second step, the cells of the second passage of the three 30 days old fetuses were seeded in two 24-well plates at a density of approximately 4 × 10^4^ and 5.5 × 10^4^ cells per well, cultured for eight days, and counted every day (3 wells each time per group) to determine the number of cells and the best population doubling time (PDT). In the third step, the cells of the second and the eighth passages were seeded in two 24-well plates at a density of approximately 4 × 10^4^ cells per well, cultured for 6 days, and counted every day (3 wells each time).

The mean cell numbers at each time point were then plotted to time using GraphPad Prism version 5.01 for Windows (GraphPad software Inc., San Diego, CA, USA). The PDT was determined based on this curve [4].

### 2.4. Cryopreservation and Reseeding

In each passage, cells at the logarithmic growth phase were collected and counted with a hemocytometer and then resuspended in frozen solution including 10% dimethyl sulfoxide (DMSO; MP Bio cat. no. 196055) and 90% FBS, at a density of 2 × 10^6^ viable cells/mL. The cell suspension was aliquoted into sterile plastic cryovials that were labeled with the fetus number, freezing serial number, and the date. The vials were sealed and kept at −20°C for 60 min to equilibrate the DMSO and then they were transferred into −70°C for 24 h, and finally transferred into liquid nitrogen for long-term storage [16]. The cryovials were removed from the liquid nitrogen and were quickly thawed in 37°C water bath. When the ice clump was almost thawed, 1 mL of cell culture medium (88% DMEM, 10% FBS, 1% penicillin and streptomycin, and 1% L-glutamine) was added, centrifuged at 1500 rpm and the cells were transferred into a flask with gently blown into uniform single cell suspension, and cultured at 37°C and 5% CO_2_.

### 2.5. Cell Viability

Viabilities before freezing and after recovery were determined using the trypan blue exclusion test (0.4% trypan blue in PBS). The number of nonviable cells was determined in a visual field of 1000 cells after one month of cryopreservation [17].

### 2.6. Karyotype Analysis

The chromosomes were prepared, fixed, and stained following standard method [18]. The cells were harvested when they reached 50–70% confluence. After hypotonic treatment, fixation, and Giemsa and Leishman staining (v : v, 1 : 3), the chromosome numbers were counted from 20 spreads under an oil immersion objective.

### 2.7. Statistical Analysis

The mean and SE of counted cells in growth curve analysis were subjected to the test of normality and then were compared using independent sample *t*-test (SPSS for Windows, version 11.5, SPSS Inc., Chicago, USA).

## 3. Results

### 3.1. Proper Age of Fetus for Fibroblast Collection

The time interval between the first and second passages for Guinea pig fetal fibroblast cells was 30 days for day 20, 10 days for day 30, and 15 days for day 45 of pregnancy. The proper pregnancy age for collection and culture of Guinea pig fetal fibroblast cells was at the 30th day of pregnancy which made the shortest interval for reaching the maximum confluence in the fibroblast cell culture.

### 3.2. Morphological Observation

**Figure 1:**
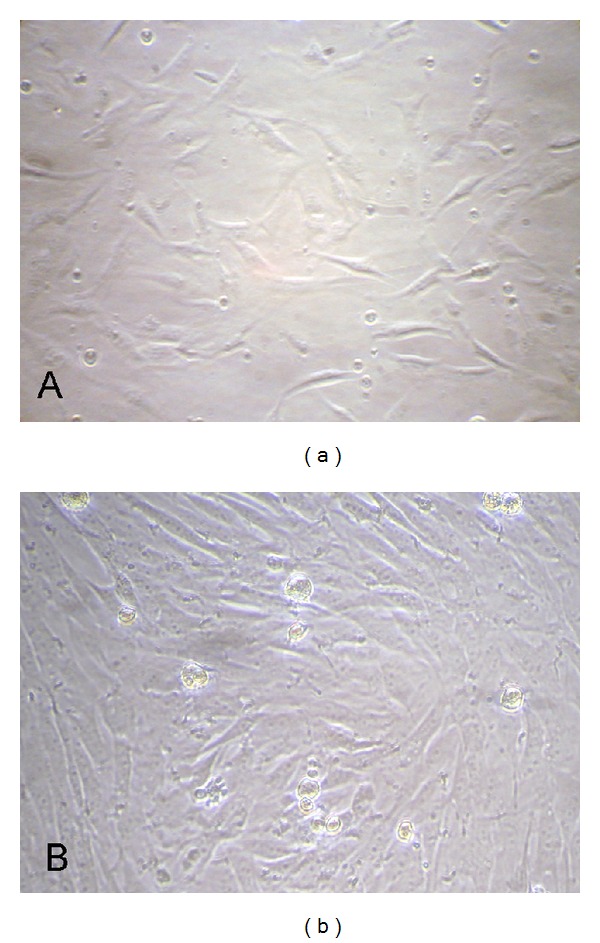
Morphology of Guinea pig fetal fibroblast cells in vitro: (a) primary cells and (b) near confluence.

At about 4-5 days after the tissue explants adhered to the flasks, fibroblast cells were observed sprouting from the margins of these tissue pieces. The cells showed typical fusiform morphology with centrally located oval nuclei (Figure 1(a)). The fetal Guinea pig fibroblast cells at the age 30 days covered the bottom of the flasks within 10 days in a monolayer shape. The cells had fibroblastic characteristics with turgor vitalis cytoplasm, and, during growth, they were morphologically fibroblast-like with radiating, flame-like, or whirlpool-like migrating patterns (Figure 1(b)).

### 3.3. Growth Curve Analysis and Cell Viabilities

**Figure 2:**
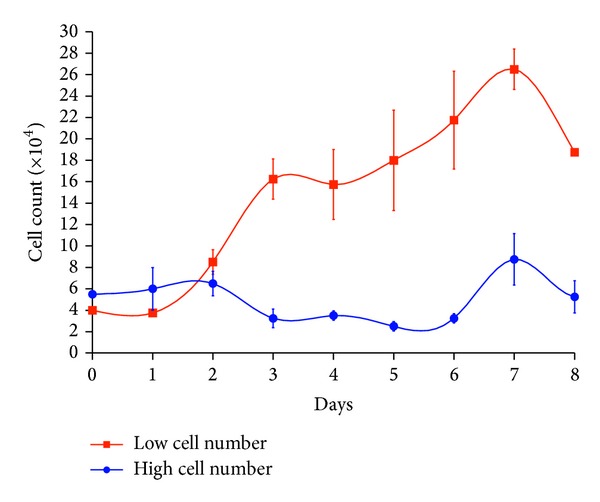
The growth curve of the second passage with initial density of approximately 4 × 10^4^ and 5.5 × 10^4^ cells per well of the Guinea pig fetal fibroblast cells (*n* = 3 fetuses and 3 counted wells/day/fetus; mean ± SE).

**Figure 3:**
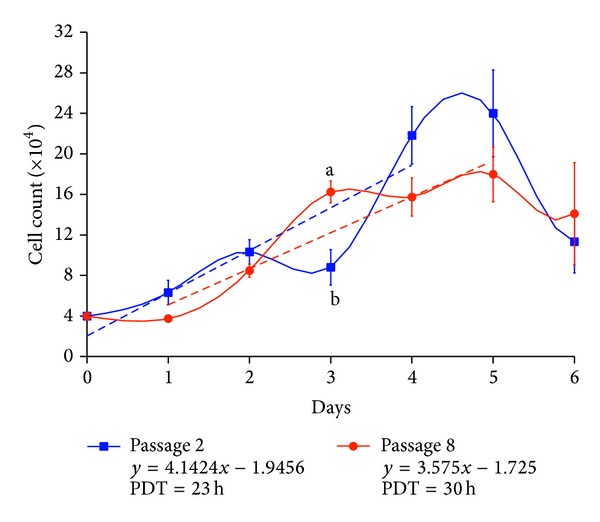
The growth curve and trend line equation of the second and eighth passages of the Guinea pig fetal fibroblast cells (*n* = 3 fetuses and 3 counted wells/day/fetus; mean ± SE).

The proper number of cells for growth curve analysis was 4 × 10^4^ per well (Figure 2). The growth curve of the Guinea pig fetal fibroblast cells exhibited a typical “S” shape and the PDT of the second passage was about 23 h and of the eighth passage was about 30 h (Figure 3). In two samples, the latent phase was about 24 h which was a result of trypsinization, followed by an exponential phase of 4 days, which gave way to stationary phase afterwards. The viability of the cultures was 95% in the second and 74.5% in the eighth passage.

### 3.4. Karyotype Analysis

**Figure 4:**
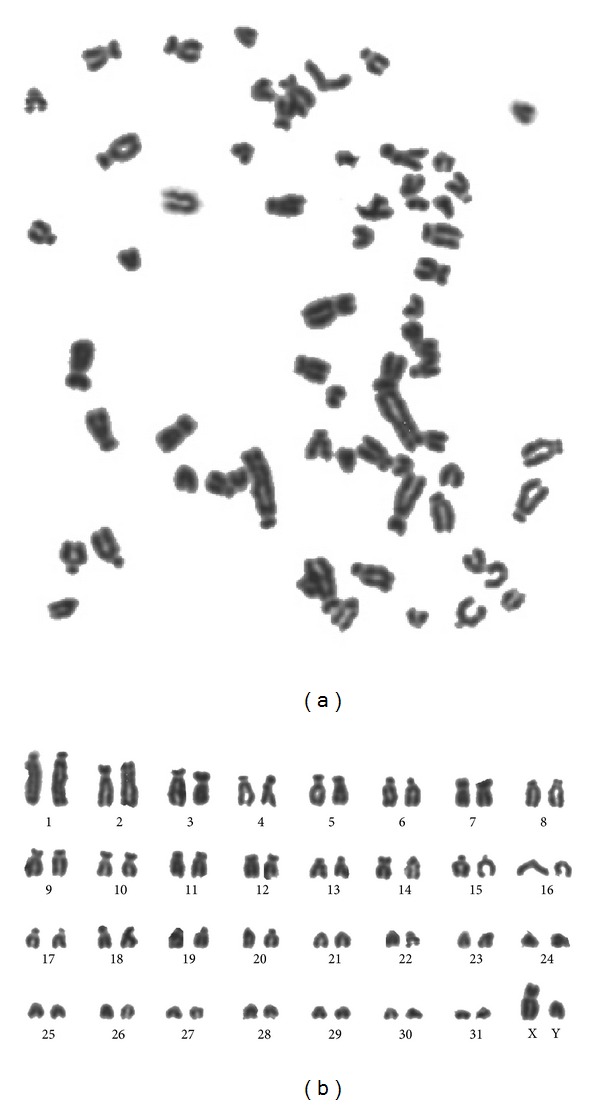
Chromosomes at metaphase (a) and karyotype (b) of the Guinea pig fetal fibroblast cells. The karyotype of the Guinea pig fetal fibroblast cells consisted of 32 pairs of chromosomes. The sex chromosome type was XY (♂).

The chromosome number of the Guinea pig fetal fibroblast cells was 2*n* = 64, comprising 62 autosomal and 2 sex chromosomes (Figure 4). The chromosome numbers per spread were counted for 20 spreads of the first passages, and the results showed that 98% of the cells were diploid, supporting the conclusion that the cell line was reproducibly diploid.

## 4. Discussion

The fetal fibroblast cell culture of the Guinea pigs was established using the adherent culture method and these cells were frozen within 8 passages. Morphology, as the most important qualitative parameter of epidermal tissue, was evaluated by light microscopy. In our study, the cells had fibrous characteristics with turgor vitalis cytoplasm, and during growth, they showed typical fibroblast-like morphology as radiating, flame-like, or whirlpool migrating shapes. Using phase-contrast microscopy, fibroblast-like, or epithelial-like cells were observed that could be seen as migrating ones from the tissue pieces 5 days after explanting [6]. When explanting tissues to derive new primary cultures, epithelial and fibroblast cells would initially grow together. Fibroblast cells adhere more easily to flasks and can be trypsinized more readily, whereas epithelial cells do not adhere in a short time and are easily shed using gentle mechanical agitation [19]. Because of these differences, fibroblast cells would quickly outgrow their epithelial counterparts. In this manner, the cells were treated with trypsin over 2 to 3 passages; then a pure fibroblast culture was obtained [20, 21].

The Guinea pig fibroblast cells were cryopreserved within 8 passages, at a density of about 2 × 10^6^ cells/mL. The viability was 95% and 74.5% after the second and eighth passages, respectively, indicating the cells were healthy with good viability under these culture conditions, while freezing resulted in damage in the cell viability after the eighth passage. The growth curve of the Guinea pig fetal fibroblast cells showed a typical “S” shape, consisting of the latent phase, exponential phase, and plateau phase. The cells after passage had a latent phase of about 24 h. Thereafter, the cells experienced exponential proliferation, followed by a plateau phase. Finally, cell growth ceased as a result of contact inhibition from the day 5. These results indicated that the second and the eighth passages of Guinea pig fetal cells cultured in vitro had the same biological characteristics of normal fibroblast cells.

Maintaining the same diploid character of fibroblast cells is critical to preserve the genetic resources in vivo. The in vitro cultured cells that kept the division capability and after successive cell divisions present any cell differentiation or alteration cannot be used in breeding conservation. A 2*n* = 64 frequency of 98% indicated that the Guinea pig fetal fibroblast cultures were stably diploid in accordance with the previous reports in Guinea pigs [22]. In addition, the frequency of diplont chromosomes was above 98%, further validating the stability of these cells. Although hypodiploid and hyperdiploid cells and some polyploid cells did emerge in the cultures with increased passaging [23], the incidence of such cells was still very small in our study (below 2%). The biological characteristics of the Guinea pig cells, especially the genetic characteristics, may change when cultured in vitro, as a result of serial passage.

It is not uncommon for cells to cease growth and show changes in biological characteristics or lose their diplont properties with time in cultures due to a variety of stimuli and factors. Effective measures are thus required to ensure diploid stability in cultures of cells that are used to preserve valuable genetic resources. The pH must therefore be controlled strictly, the culture medium must be changed at appropriate intervals, and a split ratio of between 1 : 2 and 1 : 3 should be used. Importantly, also, the passage intervals should not be too great and the cells should be maintained at subconfluence to avoid deleterious effects from contact inhibition. Explanted cells should be passaged quickly and with care and high quality reagents should always be used. Finally, cells should be cryopreserved with the utmost care as required to maintain a high quality cell bank that is viable over a long-term. The biological characteristics, especially the genetic characteristics, of the cells may be changed by in vitro culture conditions after many passages, so a minimal number of passages are recommended to conserve them [24].

In conclusion, a fibroblast cell culture was established from explanted fetal skin tissue of the Guinea pig standard tissue adherent culture and continuous passaging following trypsinization. In order to ensure the cell quality, cells of each individual were tested using the same method. We concluded that the cell quality was identical among all cell cultures. So our work made a valuable contribution to the preservation of the genetic resources of the Guinea pig and provided useful biomaterial for future studies in cell biology, medicine, genomics, postgenomics, and both genetic and embryonic engineering.
